# Death receptor 3 is involved in preeclampsia through regulating placental trophoblast cell physiology by inactivating the PI3K/AKT pathway

**DOI:** 10.1002/iid3.995

**Published:** 2023-09-27

**Authors:** Cheng Yin, Jiahui Wang, Yu Zhang, Xinping Zhang, Wei Zhao, Yanxiang Shen, Shi Liu, Su Liu

**Affiliations:** ^1^ Obstetrics Department The Third Affiliated Hospital of Qiqihar Medical University Qiqihar China; ^2^ Gynecology Department The Third Affiliated Hospital of Qiqihar Medical University Qiqihar China; ^3^ Cardiovascular Internal Medicine Department The Third Affiliated Hospital of Qiqihar Medical University Qiqihar China; ^4^ Central Laboratory The Third Affiliated Hospital of Qiqihar Medical University Qiqihar China

**Keywords:** PI3K/AKT pathway, death receptor 3, placental trophoblast cells, preeclampsia

## Abstract

**Background:**

Preeclampsia (PE) is a pregnancy related disease that affects about 5% of pregnancies. Death receptor 3 (DR3) expression is significantly elevated in both placental tissue and plasma of PE patients. However, whether DR3 was involved in trophoblasts in pathogenesis of PE are not well elucidated.

**Objective:**

Our research was designed to illustrate the biological roles of DR3 in placental trophoblasts, as well as explain its relevant mechanisms.

**Methods:**

HTR‐8/SVneo cells viability, migration, invasion, and apoptosis were assessed using MTT, Transwell assay, and flow cytometry analysis, respectively. Levels of DR3, PI3K, and AKT in HTR‐8/SVneo cells were analyzed via reverse transcription‐quantitative polymerase chain reaction assay. Western blot analysis was utilized to assess DR3, p‐PI3K, p‐AKT, PI3K, and AKT protein expression.

**Results:**

Upregulation of DR3 obviously inhibited HTR‐8/SVneo cells viability, migration, and invasion, as well as promoted HTR‐8/SVneo cells apoptosis, as opposed to the control‐plasmid group. We also found that DR3‐plasmid enhanced cleaved‐caspase3 expression, reduced p‐PI3K and p‐AKT protein expression, and p‐PI3K/PI3K or p‐AKT/AKT ratio in HTR‐8/SVneo cells. Importantly, IGF‐1, a PI3K/AKT signaling pathway agonist, partially reversed the effects of DR3‐plasmid on the cell viability, migration, invasion, apoptosis, and PI3K/AKT signal pathway in HTR‐8/SVneo cells.

**Conclusion:**

DR3 was involved in PE through regulating placental trophoblast cell physiology via PI3K/AKT pathway, which might be a promising therapeutic target for PE therapy.

## Graphical abstract

The present study was to illustrate the biological roles of death receptor 3 (DR3) in placental trophoblast cells, as well as explain its relevant mechanisms. The findings revealed that DR3 is involved in preeclampsia (PE) through regulating placental trophoblast cell physiology via PI3K/AKT pathway, which might be a promising therapeutic target for PE therapy.

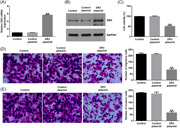

## 1 INTRODUCTION

PE, a complex disease, is a special manifestation of pregnancy induced hypertension syndrome.^1^, ^2^ The clinical manifestations are hypertension, headache, dizziness, vomiting, upper abdominal discomfort, and other symptoms, and the clinical diagnosis is mainly based on high blood pressure and proteinuria.^3^ sFlt‐1, PlGF, or its sFlt‐1/PlGF ratio can be used for assays or clinical diagnosis.^4^, ^5^ Preeclampsia (PE) etiology is complex, and multiple factors, including hypoxia, oxidative stress, and imbalance in angiogenesis, are involved in the disease mechanism. Previous studies have shown that placental dysfunction, impaired invasion of trophoblasts, abnormal remodeling of spiral arteries, and increased apoptosis of trophoblast cells are considered critical factors related to the pathogenesis of PE.^6^, ^7^ Among them, dysregulation of trophoblast cell behavior is considered important for the development of PE, and understanding the molecular mechanisms of trophoblast cell behavior may help to develop new therapeutic targets for PE.

Recent reports have revealed that the apoptosis and necrosis of cells depend on the balance between the apoptotic signaling pathway and the antiapoptotic signaling pathway.^8^, ^9^ Under pathological conditions, once this balance is broken, it will eventually lead to apoptosis.^10^ There are three pathways of apoptosis, including death receptor induced apoptosis,^11^ mitochondrial permeability induced apoptosis,^12^ and endoplasmic reticulum pathway.^13^ DR‐3, a member of TNFRSF, contains a death domain with proapoptotic effects and is able to activate caspase 8 and NF‐κB signals apoptosis by signaling cell survival.^14^ In addition, DR‐3 was verified to be closely related to the progression of many cancers, including NSCLC,^15^ breast cancer,^16^ and colon cancer.^17^ Research has shown that the expression of death receptor 3 (DR3) is significantly elevated in both placental tissue and plasma of PE patients,^18^, ^19^ and DR3 may be closely related to apoptosis of placental trophoblasts. However, the specific role and molecular regulatory mechanism of DR‐3 in PE still need further exploration. Therefore, exploring the functions of DR3 in PE is of great significance for the pathogenesis and treatment of PE.

The PI3K/AKT signaling pathway plays key roles in the regulation of cell proliferation, migration, and invasion.^20^ Previous studies have suggested that the activated PI3K/AKT pathway in PE placentas is involved in trophoblast cell proliferation.^21^, ^22^ Besides, PI3K/AKT signaling is also involved in the regulatiion of trophoblast migration and invasion.^23^, ^24^ DR3 has been reported to be the upstream of PI3K.^17^ Therefore, we hypothesized that DR3 may affect the physiology of placental trophoblasts by regulating the PI3K/AKT pathway.

Human chorionic trophoblast cells HTR‐8/SVneo has been widely used to investigate PE in vitro.^25^, ^26^ In this study, HTR‐8/SVneo was used to study the effects of DR3 on placental trophoblast cell behavior.

Thus, our research aimed to (i) explain whether DR3 was linked to the progression of PE by regulating placental trophoblast cell physiology; (ii) explore the relevance between DR3 and PI3K/AKT axis; and (iii) illustrate the mechanism of this axis in PE, as to find the promising biomarker for PE.

## 2 MATERIALS AND METHODS

### 2.1 Cell culture

HTR‐8/SVneo cells were purchased from ATCC and cultivated in RPMI‐1640 medium (Procell) containing 15% FBS and 1% penicillin/streptomycin (Procell) in a humidified incubator containing 5% CO_2_ at 37°C.

The cells were pretreated with 10 μM IGF‐1 for 30 min and then subsequent experiments were carried out.

### 2.2 Cell transfection

DR3‐plasmid or control‐plasmid was transfected into HTR‐8/SVneo cells by Lipofectamine 2000 reagent (Invitrogen) for 48 h referring to the instructions. After 48 h transfection, RNA was extracted for reverse transcription‐quantitative polymerase chain reaction (RT‐qPCR) analysis, and western blot analysis was adapted to evaluate the protein expression.

### 2.3 RT‐qPCR analysis

After treatment, the isolation of RNA from HTR‐8/SVneo cells was obtained with the TRIpure Total RNA Extraction Reagent (ELK Biotechnology) based on the protocol. Then the total RNA was reversed to cDNA following the instructions of PrimeScript RT Reagent Kit (TaKaRa) and RT‐qPCR analysis was conducted using the Enturbo^TM^ SYBR Green PCR SuperMix (ELK Biotechnology) to examine the levels of PI3K, AKT, and GAPDH. Target gene expressions were performed using $2^{-{\mathrm{\Delta \Delta C}}_{t}}$ method.

### 2.4 MTT assay

After treatment, HTR‐8/SVneo cells were implanted into 96‐well plates and treated with 10 μL MTT solution and continuously incubated for additional 4 h. Then, the supernatant was discarded and 100 μL of DMSO was added to dissolve lysate without light. Finally, OD_570_ was measured by a microplate reader (BIOTEK) following the protocol.

### 2.5 Flow cytometer (FCM) assay

After digesting the cells with trypsin without EDTA, the HTR‐8/SVneo cells were collected by centrifugation at 4°C for 5 min. After that, the cells were washed twice with PBS. For cell apoptosis assay, cells were assessed using the Annexin‐V/PI Apoptosis Detection Kit (Beyotime). The cells were gently mixed and were cultivated for 20 min at room temperature without light. Then apoptotic cells were detected by FCM (BD Technologies) and analyzed with Kaluza analysis software (v.2.1.1.20653; Beckman Coulter, Inc.).

### 2.6 Western blot analysis assay

The HTR‐8/SVneo cells were lysed using RIPA buffer (Beyotime) for 30 min and quantified by BCA Protein Assay Kit (Thermo Scientific^TM^, USA). Proteins were resolved by SDS‐PAGE and transferred onto PVDF membranes. The membranes were blocked with 5% skimmed milk for 2 h and cultivated with primary antibodies against cleaved‐caspase3, caspase3, p‐PI3K, p‐AKT, PI3K, AKT, or GAPDH (1:1000 dilution) at 4°C overnight. After washing in TBST, the membranes were cultivated with secondary antibodies for 2 h. The protein signals were assessed by ECL method following the instructions.

### 2.7 Transwell assay

Transwell chambers were precoated without or with Matrigel (BD Biosciences) to detect the abilities of migration and invasion of HTR‐8/SVneo cells, respectively. After transfection for 48 h, HTR‐8/SVneo cells were incubated in serum‐free medium for starvation and seeded into the top chamber precoated with or without Matrigel of transwell chambers, while 600 μL RPMI 1640 culture medium with 10% FBS were added to the bottom chambers. After cultivating for 48 h, the remaining cells on the top chamber were removed. Then cells adhering to the under surface of the membrane were fixed with 4% paraformaldehyde and stained with 0.1% crystal violet for 10 min. The migratory and invasive cells were counted from five random fields by an inverted microscope (Nikon).

### 2.8 Statistical analysis

Statistical analysis was conducted using GraphPad Prism 6.0 software. All results were expressed by mean ± standard deviation from three independent experiments. The statistical significance among groups were determined by one‐way ANOVA followed by Tukey's post hoc test or Student's *t*‐test. *p* < .05 indicated as statistically significant.

## 3 RESULTS

### 3.1 DR3 affected placental trophoblast cell physiology

**Figure 1:**
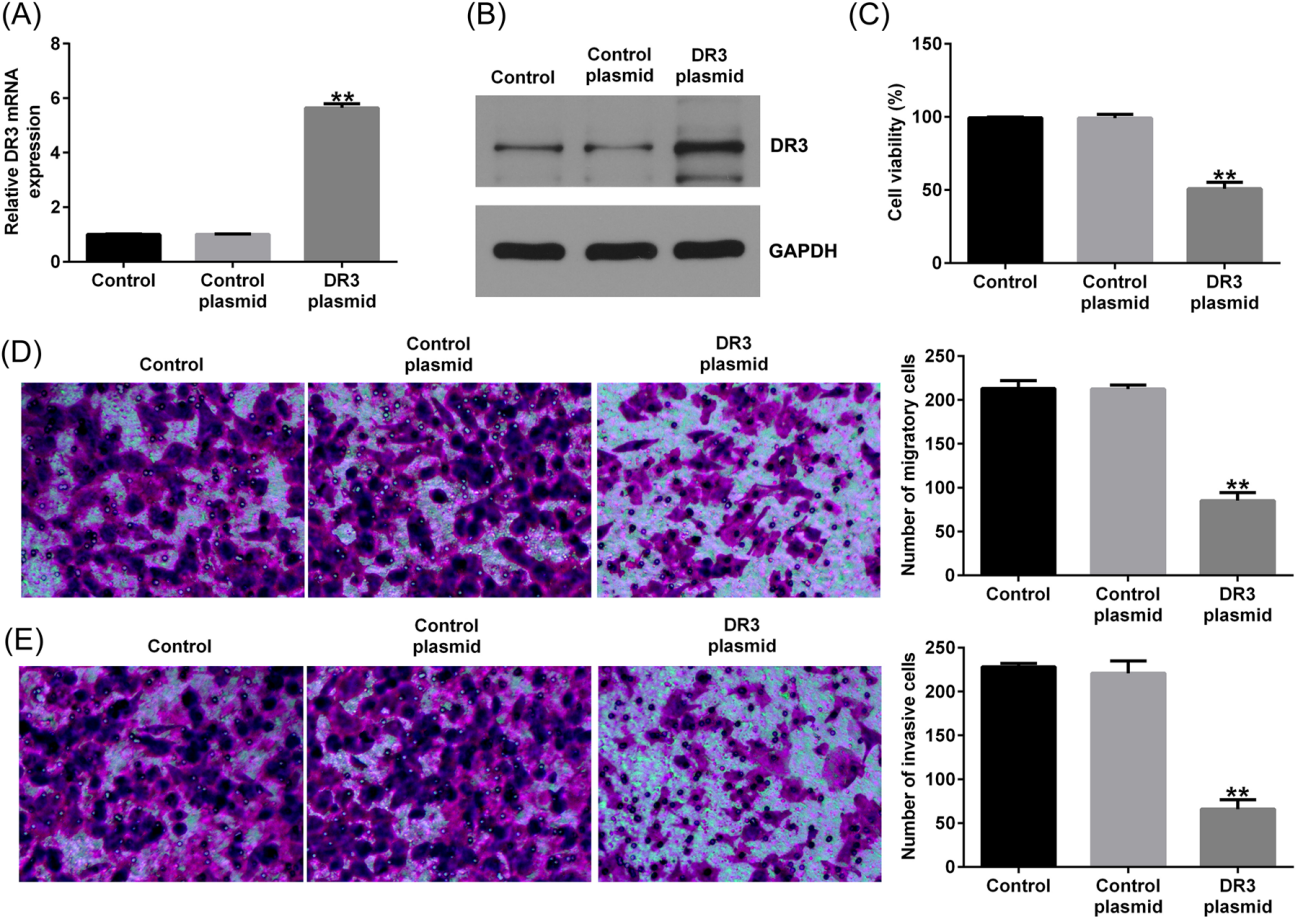
Effects of DR3‐plasmid on HTR‐8/SVneo cells proliferation, migration, and invasion. HTR‐8/SVneo cells were transfected with control‐plasmid or DR3‐plasmid for 48 h. (A, B) Detection of DR3 levels in HTR‐8/SVneo cells by RT‐qPCR and western blot analysis assay. (C) HTR‐8/SVneo cells viability was checked through MTT assay. Cells migration (D) and invasion (E) were determined through Transwell. **p < .01 versus control‐plasmid. DR3, death receptor 3; RT‐qPCR, reverse transcription‐quantitative polymerase chain reaction.

Firstly, to determine the effect of DR3 on placental trophoblast cell physiology, HTR‐8/SVneo cells were transfected with DR3‐plasmid or control‐plasmid for 48 h. Results from RT‐qPCR and western blot analysis assay suggested that DR3 levels was obviously higher in DR3‐plasmid transfected HTR‐8/SVneo cells than that in HTR‐8/SVneo cells after control‐plasmid treatment (Figure 1A,B). We also determined the effects of DR3 on HTR‐8/SVneo cells viability, migration, and invasion. As displayed in Figure 1C−E, DR3‐plasmid significantly decreased HTR‐8/SVneo cells viability, migration, and invasion.

**Figure 2:**
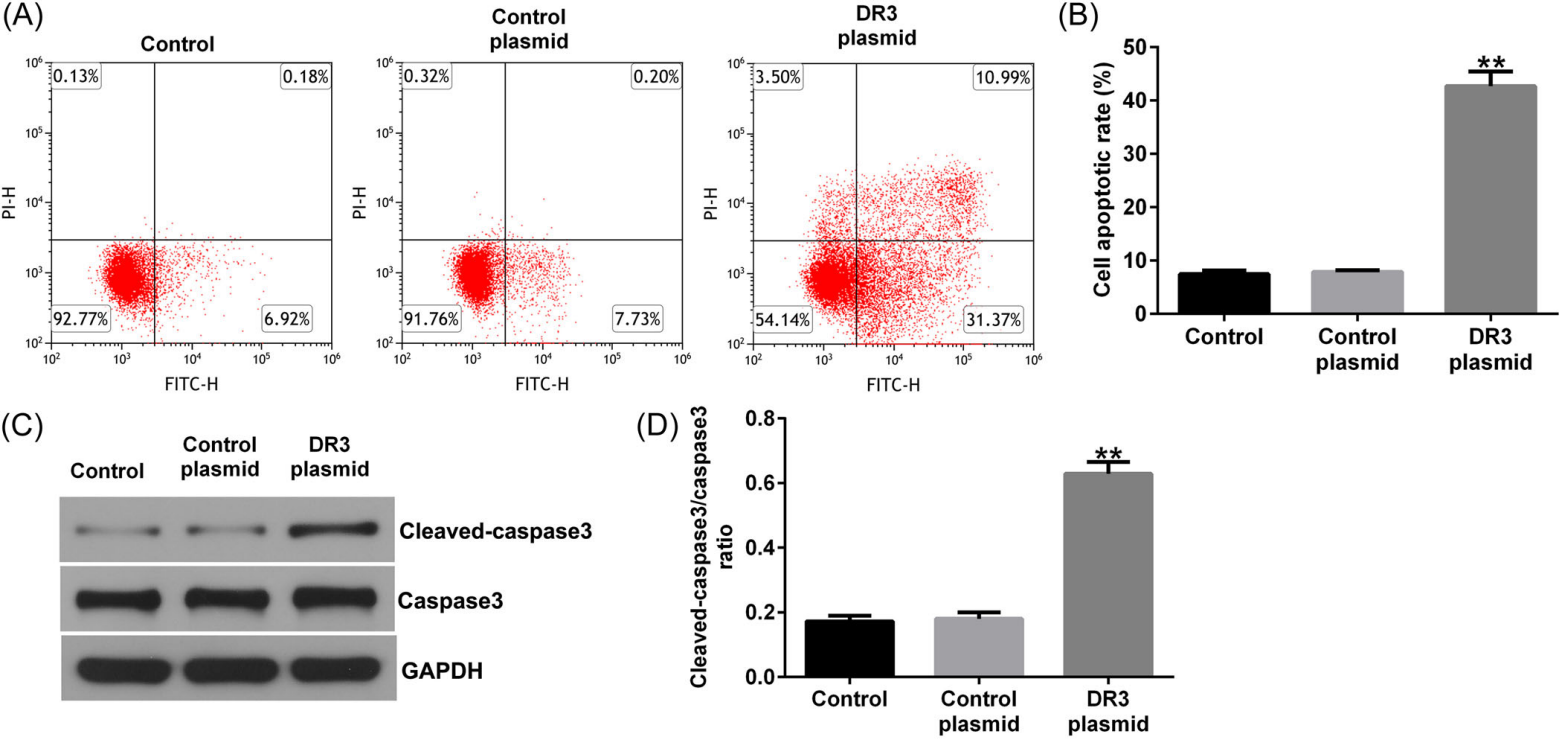
Effects of DR3‐plasmid on HTR‐8/SVneo cells apoptosis. HTR‐8/SVneo cells were transfected with control‐plasmid or DR3‐plasmid for 48 h. (A) Apoptotic cells were analyzed using FCM assay. (B) Quantitation of apoptotic cells. (C) Detection of cleaved‐caspase3 and caspase3 expression in HTR‐8/SVneo cells using western blot assay. (D) Quantification of cleaved‐caspase3/caspase3 ratio. **p < .01 versus control‐plasmid. DR3, death receptor 3; FCM, flow cytometer.

Furthermore, we illustrated the effects of DR3‐plasmid on HTR‐8/SVneo cells apoptosis. As presented in Figure 2A,B, DR3‐plasmid promoted HTR‐8/SVneo cells apoptosis. We also found that cleaved‐caspase3 and cleaved‐caspase3/caspase3 ratio was upregulated in DR3‐plasmid transfected HTR‐8/SVneo cells, as compared to control‐plasmid group (Figure 2C,D). All these results demonstrated that DR3 plays a key role in the regulation of HTR‐8/SVneo cells proliferation, apoptosis, migration, and invasion.

### 3.2 DR3‐plasmid inhibited PI3K/AKT signal pathway in HTR‐8/SVneo cells

**Figure 3:**
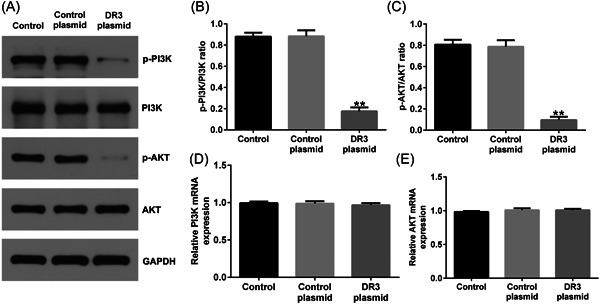
Effects of DR3‐plasmid on PI3K/AKT signal pathway in HTR‐8/SVneo cells. HTR‐8/SVneo cells were transfected with control‐plasmid or DR3‐plasmid for 48 h. (A) Western blot analysis of p‐PI3K and p‐AKT expression HTR‐8/SVneo cells. (B, C) Quantitation of p‐PI3K/PI3K and p‐AKT/AKT. (D, E) RT‐qPCR analysis of PI3K and AKT mRNA levels HTR‐8/SVneo cells. **p < .01 versus control‐plasmid. DR3, death receptor 3; RT‐qPCR, reverse transcription‐quantitative polymerase chain reaction.

We then determined the effect of DR3 on PI3K/AKT signaling pathway in HTR‐8/SVneo cells. DR3‐plasmid or control‐plasmid was transfected into trophoblast cells for 48 h. Results from western blot analysis suggested that DR3‐plasmid led to inhibiting p‐PI3K and p‐AKT expression (Figure 3A), and reduced p‐PI3K/PI3K or p‐AKT/AKT ratio (Figure 3B,C), compared to control‐plasmid group. However, there was no significant difference in the mRNA levels of PI3K and AKT among the groups (Figure 3D,E).

### 3.3 IGF‐1 reversed the effects of DR3‐plasmid on PI3K/AKT signal pathway in HTR‐8/SVneo cells

**Figure 4:**
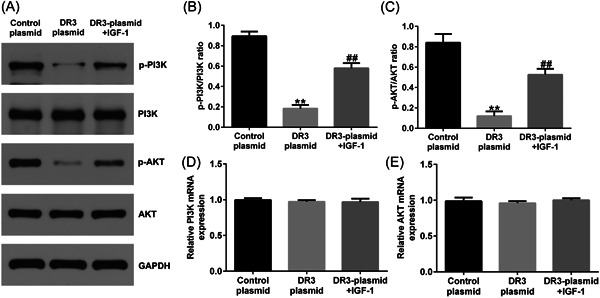
IGF‐1 reversed the effects of DR3‐plasmid on PI3K/AKT signal pathway in HTR‐8/SVneo cells. HTR‐8/SVneo cells were stimulated with 10 μM IGF‐1 for 30 min, and then transfected with control‐plasmid or DR3‐plasmid for 48 h. (A) Detection of p‐PI3K and p‐AKT expression in HTR‐8/SVneo cells. (B, C) Analysis of p‐PI3K/PI3K and p‐AKT/AKT. (D, E) mRNA levels of PI3K and AKT in HTR‐8/SVneo cells were determined using RT‐qPCR analysis. **p < .01 versus control‐plasmid; ^##^ *p* < .01 versus DR3‐plasmid. DR3, death receptor 3; RT‐qPCR, reverse transcription‐quantitative polymerase chain reaction.

To clarify whether DR3 affects HTR8/SVneo cell physiology by directly regulating the PI3K/AKT pathway, IGF1, an agonist of the PI3K/AKT signaling pathway, was used. In our research, HTR‐8/SVneo cells were stimulated with 10 μM IGF‐1 for 30 min, and then transfected with control‐plasmid or DR3‐plasmid for 48 h. Our data revealed that IGF‐1 reversed the effects of DR3‐plasmid on PI3K/AKT signal pathway, as confirmed by increased p‐PI3K and p‐AKT expression (Figure 4A), enhanced p‐PI3K/PI3K and p‐AKT/AKT ratio (Figure 4B,C), while the mRNA levels of PI3K and AKT in different groups had no obvious changes (Figure 4D,E). Our findings suggested that DR3 influences the physiology of HTR‐8/SVneo cells by regulating PI3K/AKT signaling pathway.

### 3.4 IGF‐1 reversed the effects of DR3‐plasmid on placental trophoblast cell physiology

**Figure 5:**
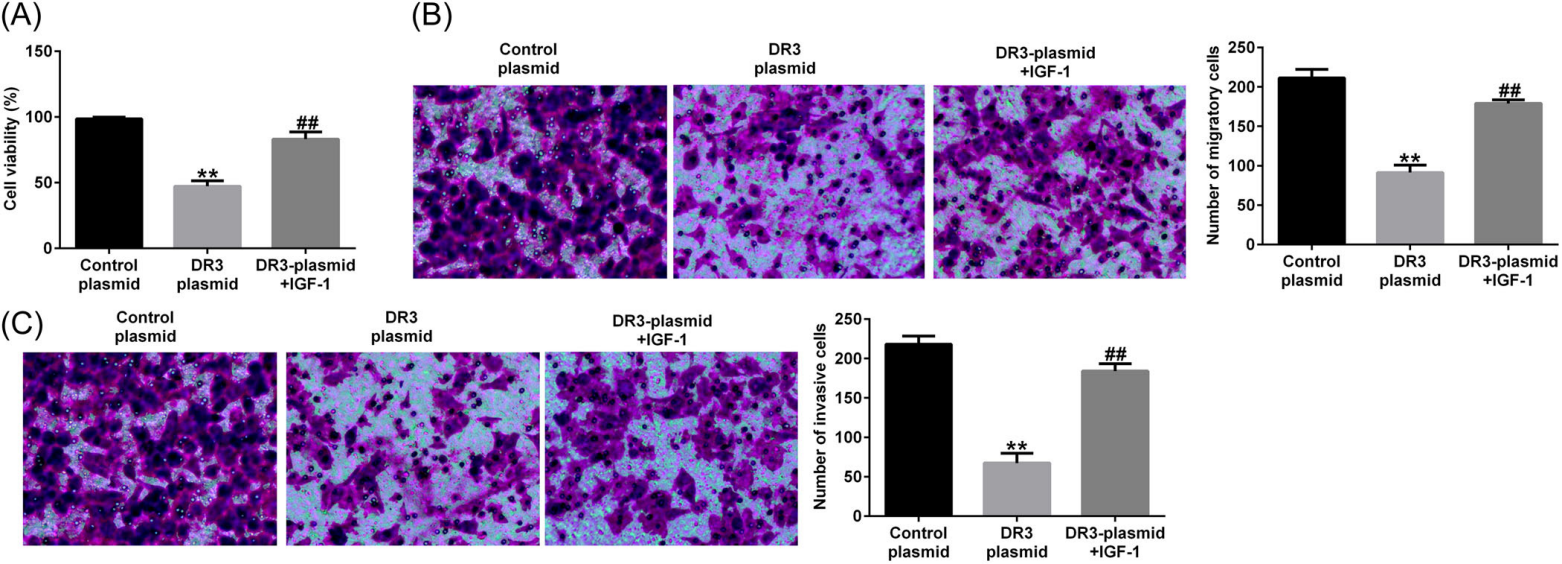
IGF‐1 reversed the effects of DR3‐plasmid on HTR‐8/SVneo cells proliferation, migration, and invasion. HTR‐8/SVneo cells were stimulated with 10 μM IGF‐1 for 30 min, and then transfected with control‐plasmid or DR3‐plasmid for 48 h. (A) MTT assay was applied for HTR‐8/SVneo cells viability detection. Cells migration (B) and invasion (C) were determined through Transwell assay. **p < .01 versus control‐plasmid; ^##^ *p* < .01 versus DR3‐plasmid. DR3, death receptor 3.

**Figure 6:**
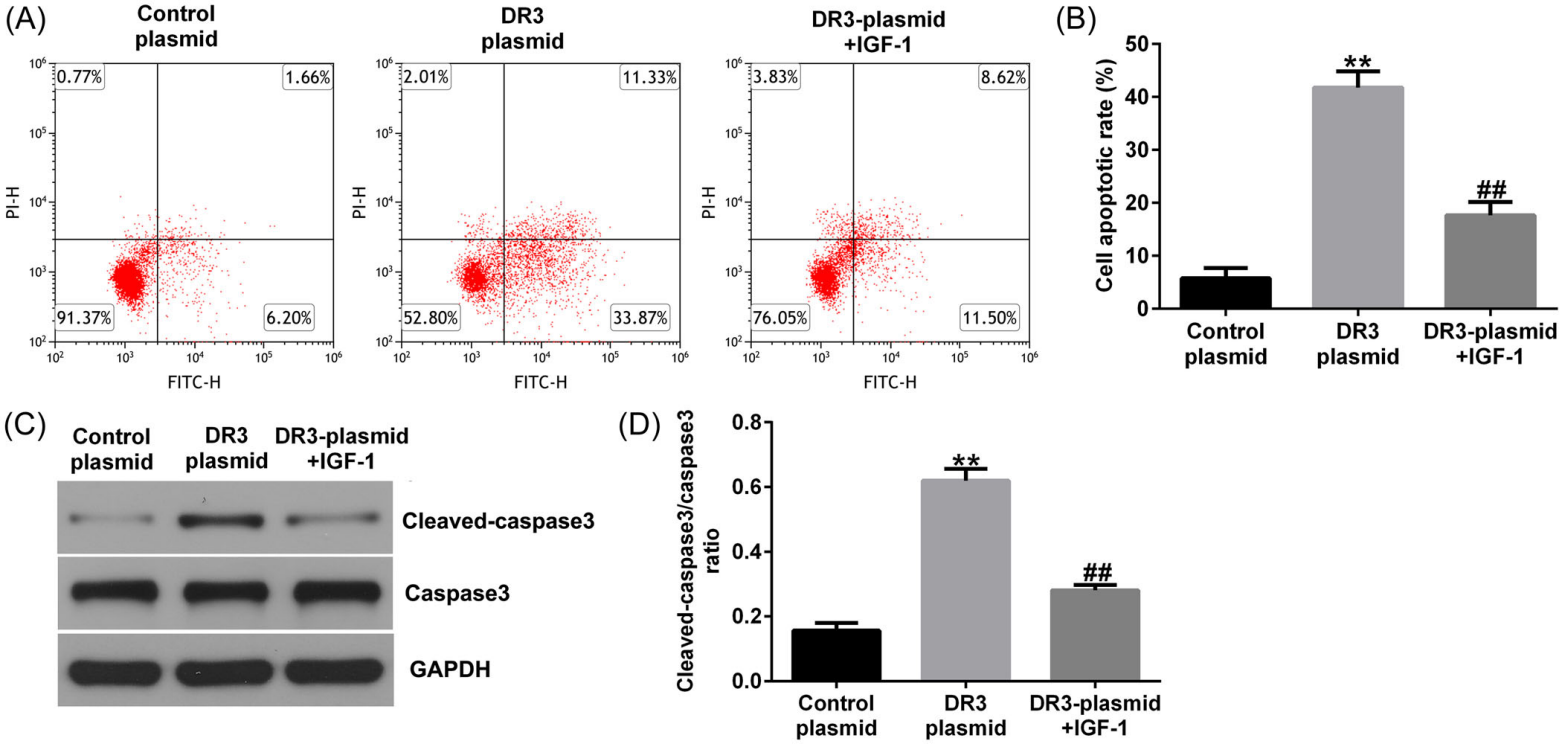
IGF‐1 reversed the effects of DR3‐plasmid on HTR‐8/SVneo cells apoptosis. HTR‐8/SVneo cells were stimulated with 10 μM IGF‐1 for 30 min, and then transfected with control‐plasmid or DR3‐plasmid for 48 h. (A) Apoptotic cells were analyzed using FCM assay. (B) Quantitation of apoptotic cells. (C) Western blot analysis of cleaved‐caspase3 and caspase3 expression in HTR‐8/SVneo cells. (D) The ratio of cleaved‐caspase3/caspase3. **p < .01 versus control‐plasmid; ^##^ *p* < .01 versus DR3‐plasmid. DR3, death receptor 3; FCM, flow cytometer.

Fianlly, we illustrated the functions of IGF‐1 in HTR‐8/SVneo cell viability, migration, invasion, and apoptosis. The data demonstrated that DR3‐plasmid inhibited cell viability (Figure 5A), reduced cell migration and invasion (Figure 5B,C). In addition, as shown in Figure 6A−D, enhanced apoptotic cells (Figure 6A,B), increased cleaved‐caspase3 expression (Figure 6C), and cleaved‐caspase3/caspase3 ratio (Figure 6D) were observed in DR3‐plasmid transfected trophoblast cells. While all these results above were eliminated by IGF‐1 treatment, demonstrating that IGF‐1 regulates HTR‐8/SVneo cells proliferation, apoptosis, and invasion through inhibiting PI3K/AKT signal pathway in PE.

## 4 DISCUSSION

PE is a pregnancy related disease that affects about 5% of pregnancies and is a major factor in maternal mortality and incidence rate worldwide.^27^, ^28^ Studies have shown that abnormal placental development in early pregnancy may be an important factor in the development of PE, including placental dysfunction,^29^ impaired trophoblast invasion,^30^ endothelial dysfunction, and increased trophoblast apoptosis.^31^ At present, hydroxychloroquine, endothelin, and phosphodiesterase inhibition are the main methods of PE treatment.^32^, ^33^, ^34^ Nevertheless, investigations on the detailed pathogenesis of PE are lacking.

Accumulating reports have verified that many genes were involved in the progression of PE. For example, Zhang et al. suggested lncRNA SNHG14 involved in trophoblast cell proliferation, migration, invasion by targeting miR‐330‐5p.^35^ Dong et al. found that Tim‐3 is correlation with PE.^36^ Syndecan 4, galectin 2, and DR3 were identified as novel proteins in pathophysiology of PE.^18^ The expression of DR3 is significantly elevated in both placental tissue and plasma of PE patients.^18^, ^19^ However, the specific functions of DR3 in trophoblasts remain unclear. Therefore, our research focus on explaining the role and mechanisms of DR3 in the trophoblast biological behaviors and searching new therapies for PE.

The abnormal regulation of HTR‐8/SVneo cells biological behaviors are considered to be vital elements in the pathogenesis of PE.^37^ Understanding the mechanism of HTR‐8/SVneo cells behavior might help us to discover novel therapeutic target for PE. In this study, we first investigated the role of DR3 overexpression (gain‐of‐function) on HTR‐8/SVneo cells, and we found that DR3 overexpression remarkably decreased HTR‐8/SVneo cells viability, migration, and invasion. Moreover, DR3 overexpression stimulated more apoptotic HTR‐8/SVneo cells, compared to control‐plasmid group. Caspase3, a member of caspase family, was evidenced to be a vital regulator in cells apoptosis.^38^ We also determined the status of caspase3 in HTR‐8/SVneo cells, and the findings indicated that DR3‐plasmid significantly upregulated cleaved‐caspase3 levels and cleaved‐caspase3/caspase3 ratio in HTR‐8/SVneo cells, compared to control‐plasmid group. These findings suggested that DR3‐plasmid remarkably inhibited trophoblast cells growth and invasion, and stimulated apoptosis, suggesting its important role in trophoblast invasion and apoptosis in PE.

PI3K/AKT signal pathway has been reported to play an important role in regulating various cell functions, including growth, proliferation, survival, transcription, and protein synthesis.^39^, ^40^ We also illustrated the relationship between DR3 and PI3K/AKT signaling pathway in HTR‐8/SVneo cells in this study. According to western blot analysis, we observed that DR3‐plasmid inhibited PI3K/AKT signal pathway in HTR‐8/SVneo cells. In vitro observations have demonstrated that initiation of PI3K/AKT pathway by IGF‐1 decreases spinal cord injury‐induced endothelial apoptosis and microvascular damage.^41^ Javvaji et al. have confirmed that IGF‐1 treatment improves developmental potential of ovine oocytes through the regulation of PI3K/AKT and apoptosis signaling.^42^ In this research, to clarify whether DR3 affects HTR8/SVneo cell physiology by directly regulating the PI3K/AKT pathway, IGF1, an agonist of the PI3K/AKT signaling pathway, was used. HTR‐8/SVneo cells were stimulated with 10 μM IGF‐1 for 30 min, and then transfected with control‐plasmid or DR3‐plasmid for 48 h. We found that IGF‐1 significantly reversed the effects of DR3‐plasmid on PI3K/AKT signal pathway, HTR‐8/SVneo cell viability, migration, and invasion, suggesting that DR3 influences the physiology of HTR‐8/SVneo cells by regulating PI3K/AKT signaling pathway.

There were also some limitations of this study. First, the loss‐of‐function of DR3 in HTR‐8/SVneo cells was not performed in this research. Second, this study did not delve into the downstream and upstream signaling molecules (such as BCAP, GSK‐3β, and mTOR) and pathways (JNK and p53 pathways) for PI3K/AKT pathway in HTR‐8/SVneo cells. Also, the relationship between DR3 and IGF1 was not investigated in the present study. In addition, the role of DR3 in PE was not investigated in PE animal models. We will perform these issues in the future.

## 5 CONCLUSION

Our results shed some light on the progression of PE, shown that DR3 regulates trophoblast cells physiology via PI3K/AKT signal pathway in PE, which may be a novel molecular therapeutic target for PE therapy.

## AUTHOR CONTRIBUTIONS

**Cheng Yin**: Conceptualization (lead); writing—original draft (lead); investigation (lead); formal analysis (lead); writing—review and editing (equal). **Jiahui Wang, Yu Zhang, and Xinping Zhang**: Software (equal); methodology (equal); investigation (equal). **Wei Zhao, Yanxiang Shen, Shi Liu, and Su Liu**: Methodology (equal); investigation (equal).

## CONFLICT OF INTEREST STATEMENT

The authors declare no conflict of interest.

## Data Availability

The data sets used and/or analyzed during the current study are available from the corresponding author on reasonable request.
